# Hyperkalaemia in Heart Failure: Consequences for Outcome and Sequencing of Therapy

**DOI:** 10.1007/s11897-022-00552-3

**Published:** 2022-06-15

**Authors:** Daniel Murphy, Debasish Banerjee

**Affiliations:** 1 Institute of Medical and Biomedical Education, St George’s, University of London, Cranmer Terrace, London, SW17 0RE UK; 2 Renal and Transplantation Department, St George’s, University Hospitals NHS Foundation Trust, Blackshaw Road, London, SW17 0QT UK

**Keywords:** Hyperkalaemia, Heart failure, Hyperkalaemia management, RAASi, Potassium binder

## Abstract

**Purpose of Review:**

Heart failure (HF), in conjunction with common comorbidities such as chronic kidney disease and diabetes and medical therapies such as RAASi, predisposes to hyperkalaemia which may lead to hospitalisation and death. This paper aims to review the most current evidence surrounding the risks and management of hyperkalaemia in HF, with particular focus on recent research into RAASi including novel selective mineralocorticoid receptor blockers and novel potassium binders.

**Recent Findings:**

The most recent evidence shows that even moderate hyperkalaemia may predispose to adverse outcomes such as hospitalisation and death. Furthermore, it may prevent patients from receiving optimal medical therapy for HF by reducing prescription of RAASi therapy. Novel potassium binders such as sodium zirconium cyclosilicate (SZC) and patiromer present potential options to reduce and prevent hyperkalaemia as well as maintain optimal RAASi dosing in HF.

**Summary:**

Management of hyperkalaemia in HF has advanced in recent years. New therapies such as SZC and patiromer are contributing to the management of acute hyperkalaemia and also access to life-saving RAASi therapies by tackling and preventing hyperkalaemia in the community.

## Introduction

Cardiovascular diseases, including heart failure, are a leading cause of mortality across the world and contribute massively to global disability burden [1]. Heart failure itself, comorbid conditions such as diabetes mellitus (DM) and chronic kidney disease (CKD), and medical therapies for heart failure such as renin–angiotensin–aldosterone system inhibitors (RAASi) all predispose to hyperkalaemia, which in turn may lead to emergency hospitalisation and death. Indeed, one recent study estimates that almost one-quarter of heart failure patients experience at least one episode of hyperkalaemia each year [2]. Maintaining normal serum potassium concentrations (sK^+^) in individuals with heart failure is therefore a major clinical challenge. This review will discuss the challenge of hyperkalaemia in heart failure, briefly covering the physiology of hyperkalaemia before focussing on clinical outcomes and management options. In particular, the most recent evidence regarding the risks of hyperkalaemia, its impact on RAASi dosing, and the impact of novel potassium binders such as patiromer and sodium zirconium cyclosilicate will be discussed.

## Potassium Homeostasis

Around 98% of the body’s K^+^ content exists within the intracellular compartment, with just 2% found in the extracellular fluid. Normal sK^+^ of around 3.5–5.0 mmol/L is maintained by several mechanisms: gut absorption of ingested potassium, movement of K^+^ between compartments, excretion via the kidneys, and to a limited extent by gastrointestinal excretion. Following a meal, insulin stimulates cellular uptake of K^+^ by increasing the activity of the Na^+^/K^+^-ATPase. Similarly, catecholamines acting at β_2_-adrenergic receptors stimulate cellular K^+^ uptake via the Na^+^/K^+^-ATPase.

The vast majority of K^+^ excretion (90%) occurs in the kidneys and is regulated by the renin–angiotensin–aldosterone (RAA) axis. K^+^ is freely filtered by the glomerulus and is resorbed in the proximal tubule via the paracellular pathway and in the thick ascending limb of the loop of Henle via both the paracellular route and the Na^+^-K^+^-2Cl^−^ transporter. Hence, only a small proportion of filtered K^+^ reaches the site of action of the RAA axis at the distal tubule. In the early distal tubule, Na^+^ is resorbed via the thiazide-sensitive Na^+^/Cl^−^ cotransporter (NCC). Increasing extracellular K^+^ concentrations in the renal interstitium downregulate NCC activity, increasing the amount of Na^+^ reaching the late distal tubule. Here, and in the collecting duct, Na^+^ is resorbed via epithelial Na^+^ channels (ENaC) and K^+^ is secreted down an electrical gradient via renal outer medullary K^+^ (ROMK) and big K^+^ (BK) channels. Under the normal action of the RAA axis, aldosterone acts at mineralocorticoid receptors in the distal tubule and collecting duct epithelial cells to upregulate ENaC, leading to increased Na^+^ resorption and K^+^ secretion. Inhibition of the RAA axis, particularly via the use of mineralocorticoid receptor antagonists (MRAs), angiotensin-converting enzyme inhibitors (ACEis), and angiotensin receptor blockers (ARBs), may hence reduce K^+^ excretion by acting here.

Interestingly, recent evidence has emerged to suggest an aldosterone-independent mechanism of K^+^ excretion centred around gut–kidney communication [3]. Here, the authors demonstrated that, following a K^+^-deficient complex meal combined with a 35 mmol oral K^+^ load, there was a sharp rise in K^+^ excretion despite MR blockade with eplerenone. This rise in K^+^ excretion did not occur when participants were given the oral K^+^ load only, suggesting the presence of a gut–kidney signalling axis for K^+^ excretion.

## Mechanisms of Hyperkalaemia

In heart failure, hyperkalaemia may be caused by RAASi medication, by heart failure itself, or by comorbidities such as DM or CKD. ACEi and ARB induce hyperkalaemia via renal vasoconstriction, effectively reducing glomerular filtration rate (GFR) and hence the amount of K^+^ filtered, and by reducing aldosterone production. Lower GFRs also result in less Na^+^ being delivered to the distal nephron, so less Na^+^–K^+^ exchange can take place. As mentioned, MRA medications block the action of aldosterone at the distal nephron, reducing Na^+^ resorption and hence reducing K^+^ secretion.

Heart failure may also induce hyperkalaemia per se. Effective renal hypoperfusion secondary to impaired cardiac output leads to reduced GFR, hence reducing K^+^ secretion as outlined above. CKD increases the risk of hyperkalaemia through reduced GFR. DM may increase risk of hyperkalaemia through damage to the kidney, resulting in a state of hyporeninaemic hypoaldosteronism which limits K^+^ secretion, and was associated with hazard ratios (HRs) of 1.33 for hyperkalaemia in an analysis of the Swedish HF Registry and 1.38 in a Danish study [2, 4, 5]. Interestingly, in the Swedish analysis, haemoglobin concentration < 120 g/L was associated with a HR of 1.43 for hyperkalaemia [2].

## Outcomes in Hyperkalaemia

Hyperkalaemia can lead to a range of negative outcomes including emergency hospitalisation, arrhythmia, and death. In a 2019 systematic review, Palaka et al. assessed outcomes stratified by degree of hyperkalaemia [6]. The authors demonstrated that sK^+^ 5.5–6.0 mmol/L in patients with HF carried HRs of 2.94 for all-cause and 1.88 for cardiovascular mortality compared to normokalaemic patients. sK^+^  > 6.0 mmol/L carried HRs of 4.89 and 3.33 for all-cause and cardiovascular mortality, respectively. Perhaps unsurprisingly, risk of cardiovascular mortality for a given sK^+^ was higher for individuals with HF than for individuals with CKD.

While the review used sK^+^  > 5.5 mmol/L as its definition of hyperkalaemia, the authors noted that 18 of 67 studies included used a threshold of 5.0 mmol/L. Indeed, sK^+^ of 5.0–5.5 mmol/L in HF patients carried HRs of > 2 and > 1 for all-cause and cardiovascular mortality, respectively. A subsequent study based on the EPHESUS cohort produced a risk tool for HF patients which showed that sK^+^ 5.1–5.5 mmol/L carried HRs of 1.3 for cardiovascular mortality and 1.0 for HF hospitalisation, while sK^+^  > 5.5 mmol/L carried HRs of 2.1 and 2.2, respectively [7]. This suggests that though sK^+^  > 5.5 mmol/L or > 6.0 mmol/L seem to carry the highest risk, even high-normal sK^+^ levels could predispose to mortality and hospitalisation in HF patients, indicating the need for close monitoring and control.

In acute heart failure following a first myocardial infarction, evidence suggests that a single episode of hyperkalaemia is associated with increased risk of all-cause mortality in the first 90 days, with HRs of 2.0 and 5.6 for sK^+^ of 5.1–5.5 mmol/L and > 5.5 mmol/L, respectively [8].

There is also evidence that episodes of hyperkalaemia may predict progression to CKD. A prospective 2018 study of 2443 patients demonstrated that presence of hyperkalaemia in either both or the second of two clinic visits in a 12-month period predicted progression to end-stage kidney disease (ESKD) independent of changes in patients’ eGFR [9]. While this cohort was not specific for heart failure, 36% of patients overall had cardiovascular disease (CVD).

## RAASi and Hyperkalaemia

The European Society of Cardiology recommends RAASi treatment for all patients with heart failure with reduced ejection fraction (HFrEF) to reduce mortality, prevent hospitalisation, and improve functional status [10]. However, RAASi medications predispose to hyperkalaemia, limiting their use, especially in individuals with comorbidities such as CKD. As such, one of the first actions clinicians may take for a hyperkalaemic patient, especially in an acute setting, is to stop RAASi medications. Incident hyperkalaemia in patients hospitalised with heart failure has been associated with long-term down-titration of MRA medications, and individuals who have their MRA medications down-titrated had higher mortality rates within 180 days compared with individuals who did not have their MRAs altered [11]. A recent study of over 434,000 RAASi users found an incidence of 1.3 episodes of hyperkalaemia per 100 patient-years, and HRs for interruption and cessation of therapy of 1.1 and 3.4 following severe hyperkalaemia, respectively [12]. Risks of interruption of RAASi therapy were higher for patients with CKD and HF. Another study found relative risk of RAASi interruption following an event of sK^+^ 5.5–6.0 mmol/L to be 1.32, and for sK^+^  > 6.0 mmol/L to be 2.19 [13]. Hence, we can see that incident hyperkalaemia poses a significant challenge to the maintenance of RAASi therapy.

A 2019 study of Danish population registry data aimed to quantify predictors of hyperkalaemia in patients receiving first-time RAASi prescription, CKD, or HF [14]. The authors identified that, following a first episode of hyperkalaemia, 37% of RAASi users, 40% of CKD patients, and 49% of HF patients had a further episode within six months. Further, they found that low eGFR, diabetes, and spironolactone use were independent risk factors for repeat hyperkalaemia.

Clinical trials examining the effects of RAASi medications report the incidence of hyperkalaemia in their cohorts; these are summarised in Table 1. The majority of trials demonstrate a higher incidence of hyperkalaemia in groups receiving RAASi therapy. The PARADIGM-HF trial of valsartan/sacubitril vs enalapril [15] and the PARAGON-HF trial of valsartan/sacubitril vs valsartan alone [16] demonstrated lower rates of hyperkalaemia in their intervention arms compared to controls; however, the PIONEER-HF trial of valsartan/sacubitril vs enalapril in acute decompensated HF demonstrated higher rates of hyperkalaemia in the intervention group [17]. There is inconsistent reporting of deaths and hospitalisations related to hyperkalaemia in RAASi trials. The EMPHASIS-HF trial reported zero deaths from hyperkalaemia with four hyperkalaemia hospitalisations in the treatment group and three in the control group [18]. Similarly, the TOPCAT and ALBATROSS trials reported zero deaths from hyperkalaemia, but did not report specific hospitalisation numbers [19, 20].

**Table 1:** Clinical trials examining the effects of RAASi medications. *eGFR* estimated glomerular filtration rate, *IQR* interquartile range, *NR* not reported, *SD* standard deviation, *sK*^+^ serum K^+^

Trial	Year	Sample size	Intervention	Mean (± SD) eGFR (mL/min/1.73 m^2^)	Incidence of hyperkalaemia	Mean change in sK^+^	Mean change in creatinine
SOLVD [21]	1991	2,569	Enalapril vs placebo	Mean creatinine 106.1 mmol/L both groups	Defined as > 5.5 mmol/L. 6.4% in intervention vs 2.5% in control group	Intervention group higher than control group by 0.2 mmol/L	+ 8.8 mmol/L in the intervention group
RALES [22]	1999	1,663	Spironolactone vs placebo	NR	Defined as > 6.0 mmol/L. 1.7% in intervention vs 1.2% in control group	+ 0.3 mmol/L in intervention group. No change in control group	+ 4–9 mmol/L in intervention group. No change in control group
ValHeFT [23]	2001	5,010	Valsartan vs placebo	NR	NR	+ 0.12 mmol/L in intervention vs –0.07 mmol/L in control group	+ 15.9 mmol/L in intervention vs + 8.8 mmol/L in control group
CHARM [24–26]	2003	4,576	Candesartan ± ACE-I vs placebo	NR	Discontinuation due to hyperkalaemia: 2.8% in intervention vs 0.5% in control group	NR	Discontinuation due to rising creatinine: 7.1% in intervention vs 3.5% in control group
EMPHASIS-HF [18]	2011	2,737	Eplerenone vs placebo	71.2 ± 21.9 in intervention vs 70.4 ± 21.7 in control group	> 5.5 mmol/L: 11.8% in intervention vs 7.2% in control group. > 6.0 mmol/L: 2.5% in intervention vs 1.9% in control group	+ 0.16 mmol/L in intervention group vs + 0.05 mmol/L in control group	+ 8.0 mmol/L in intervention group vs + 3.5 mmol/L in control group
PARADIGM-HF [15]	2014	8,442	Valsartan/ sacubitril vs enalapril	NR	> 5.5 mmol/L: 16.1% in intervention vs 17.3% in control group. > 6.0 mmol/L: 4.3% in intervention group vs 5.6% in control	NR	Mean change NR. Incidence > 221 mmol/L 139 in intervention group vs 188 in control group
TOPCAT [27]	2014	3,445	Spironolactone vs placebo	Median (IQR): 65.3 (53.9–79.2) in intervention vs 65.5 (53.5–79.1) in control group	Defined as > 5.5 mmol/L. 18.7% in intervention vs 9.1% in control group	NR	Mean change NR. Creatinine doubled to value above normal range in 10.2% in intervention group vs 7.0% in control
ALBATROSS [20]	2016	1,603	Spironolactone + standard care vs standard care post-MI	Creatinine clearance, median (IQR): 96.0 (75.4–119.7) mL/min in intervention vs 101.4 (77.3–121.1) mL/min in control group	Defined as > 5.5 mmol/L. 3% in intervention vs 0.2% of control group	NR	NR
PARAGON-HF [28]	2019	4,882	Valsartan/ sacubitril vs valsartan	63 ± 19 in intervention vs 62 ± 19 in control group	> 5.5 mmol/L: 13.2% in intervention vs 15.2% in control group. > 6.0 mmol/L: 3.1% in intervention vs 4.6% in control group	NR	Mean change NR. Incidence > 221 mmol/L 261 (10.8%) in intervention group vs 328 (13.7%) in control group
PIONEER-HF [29]	2019	881	Valsartan/ sacubitril vs enalapril in acute HF	Median (IQR): 58.4 (47.5–71.5) in intervention vs 58.9 (47.4–70.9) in control group	Defined as > 5.5 mmol/L. 11.6% in intervention vs 9.3% in control group	Graph in Supplementary Appendix but values NR	Graph in Supplementary Appendix but values NR. Increase > 44 mmol/L: 13.6% in intervention vs 14.7% in control group
FIDELIO-DKD [30]	2021	5,674	Finenerone vs placebo	44.4 ± 12.5 in intervention vs 44.3 ± 12.6 in control group	> 5.5 mmol/L: 21.7% in intervention group vs 4.5% in control. > 6.0 mmol/L: 9.8% in intervention group vs 1.4% in control	Intervention group higher than control group by 0.23 mmol/L at month four	eGFR reported
FIGARO-DKD [31]	2021	7,437	Finenerone vs placebo	67.6 ± 21.7 in intervention vs 68.0 ± 21.7 in control group	> 5.5 mmol/L: 13.5% in intervention group vs 6.4% in control. > 6.0 mmol/L: 2.3% in intervention group vs 1.2% in control	Intervention group higher than control group by 0.16 mmol/L	Graph in Supplementary Appendix but values NR

The FIDELIO-DKD and FIGARO-DKD trials reported 40 and 21 hospitalisations in their treatment groups compared with 8 and two hospitalisations in their control groups, respectively [30, 31]. In the FIDELIO-DKD trial, sK^+^  > 5.5 mmol/L occurred in 21% and lower eGFR, higher urine albumin, beta-blocker were associated with higher risk [32]. These trials focussed on a combined CKD-HF population with average eGFRs of 44 mL/min/1.73 m^2^ and 68 mL/min/1.73 m^2^, in FIDELIO-DKD and FIGARO-DKD, respectively, which may help to explain both the rates of hospitalisation and the authors’ acknowledgement of reporting them.

## Newer Therapies and Hyperkalaemia

A major shift in prescribing for HF has occurred following recent trials of SGLT2 inhibitors. Evidence has shown that SGTL2 inhibitors such as dapagliflozin and empagliflozin improve outcomes in HF patients even in the absence of diabetes [33, 34], and recent guidelines recommend prescription of SGLT2 inhibitors in addition to standard HF therapies [35, 36]. Current evidence suggests that these therapies do not worsen renal function. Large trials of SGLT2 inhibitors have shown no significant changes in eGFR versus placebo [33, 34, 37, 38]. There is evidence of a short-term, reversible drop in glomerular filtration on initiation of SGLT2 inhibitors owing to their action in reducing glomerular pressure; this action translates into longer-term protection of renal function [39–41].

Large trials of SGLT2 inhibitors in HF have not often reported hyperkalaemia rates specifically. However, secondary analysis of the EMPEROR-Reduced trial showed reduced rates of hyperkalaemia and reduced MRA discontinuation in patients taking MRA and SGLT2i versus MRA and placebo [42]. In addition, evidence from trials in individuals with diabetes suggests that SGLT2 inhibitors may reduce incidence of hyperkalaemia [43–45], indicating a favourable safety profile in this respect. There is therefore no current evidence to discourage the use of potentially beneficial SGLT2 inhibitors for fear of hyperkalaemia.

Vericiguat is a soluble guanylate cyclase stimulator which was approved in the EU and USA in 2021 for use in individuals with HFrEF who have recently required intravenous management, and is endorsed for this use by the 2021 ESC Heart Failure Guidelines [35]. It was evaluated in the large VICTORIA trial, demonstrating a primary end-point (hospitalisation for HF or cardiovascular death) event rate of 35.5% compared with 38.5% in the placebo group over a median follow-up of 11 months [46]. The trial showed a lower rate of hyperkalaemia in the vericiguat group (4.4% vs 5.6% in the placebo group), but also showed a higher rate of anaemia in the treatment arm (7.6% vs 5.7%). Vericiguat therefore represents an option for patients at high risk of recurrent hospitalisation due to HF, though the authors’ sub-group analysis showed no significant reduction in primary outcomes for individuals with eGFR ≤ 30 mL/min/1.73 m^2^ or > 60 mL/min/1.73 m^2^.

Omecamtiv mecarbil, a cardiac-specific myosin activator, demonstrated a modest reduction in heart failure events and cardiovascular deaths in individuals with NYHA class III/IV HF in the recent GALACTIC-HF trial [47]. This beneficial impact on individuals with severe HF, but not non-severe HF, was supported in a post hoc analysis published this year [48]. Both the original GALACTIC-HF trial data and the post hoc analysis showed no changes in sK^+^ or serum creatinine in the intervention versus placebo groups. Omecamtiv mecarbil is not currently licensed for HF.

## Managing Hyperkalaemia

Management of hyperkalaemia in individuals with HF can be divided into acute management, management after an acute episode, and chronic management. In the last few years, novel therapies such as patiromer and sodium zirconium cyclosilicate have begun to enter clinical practise for the management of acute and chronic hyperkalaemia [49, 50]. Before discussing management strategies, we will provide a brief overview of these novel therapies, their benefits, and potential disadvantages.

Patiromer is a non-absorbed cation exchange polymer which acts to bind K^+^ in the gut lumen in exchange for Ca^2+^, therefore reducing intestinal absorption of K^+^ while increasing intestinal absorption of Ca^2+^. It is administered as a dispersible oral powder in doses of 8.4 g up to a maximum of 25.2 g per day. It was approved for use in the management of hyperkalaemia by the US Food & Drug Administration (FDA) in 2015 and the European Medicines Agency (EMA) in 2017. It has been shown in clinical studies to be effective in reducing sK^+^ and enabling use of RAASi medications in non-acute settings [51–53]. Although the quoted onset of action for patiromer is 4–7 h [54], pilot data from a 2019 study in 30 patients with ESKD showed that treatment with patiromer plus standard care resulted in reduced sK^+^ two hours after treatment compared to standard care alone, though there was no difference between treatment groups at six hours [55]. A recent meta-analysis reported no significant difference in adverse event rates between patiromer and placebo groups in the literature [56].

Sodium zirconium cyclosilicate (SZC) is also a non-absorbed cation exchange polymer but binds K^+^ in the gut lumen in exchange for Na^+^. It was approved for use by the FDA and EMA in 2018 and is administered orally as a dispersible powder, initially at 10 g three times per day for 72 h, then continuing at a lower maintenance dose titrated according to sK^+^. Large-scale evidence for the use of SZC in hyperkalaemia has emerged over the last decade, demonstrating that SZC was effective at restoring and maintaining sK^+^ in patients, including those with CKD and HF [57–60]. In particular, a phase two study by Ash et al. demonstrated that SZC was as effective at controlling sK^+^ in individuals taking RAASi therapy as in those who were not [57]. In addition, a sub-group analysis of the HARMONIZE trial demonstrated that SZC was effective in normalising sK^+^ and maintaining RAASi dosing in HF patients [61]. Similarly to patiromer, recent evidence has shown that SZC is effective as an adjunctive treatment for hyperkalaemia in the acute setting. Participants presenting to the emergency department with sK^+^  ≥ 5.8 mmol/L who were randomised to SZC plus standard care had 0.35 mmol/L lower sK^+^ at two hours and 0.13 mmol/L lower at four hours compared to placebo plus standard care [62]. While the large-scale HARMONIZE-Global study [60] reported oedema and constipation as more common in those taking SZC compared to placebo, recent meta-analysis suggests that while oedema is more common in individuals taking SZC, there is no significant difference in rates of constipation [56].

## Acute Management of Hyperkalaemia

**Fig. 1:**
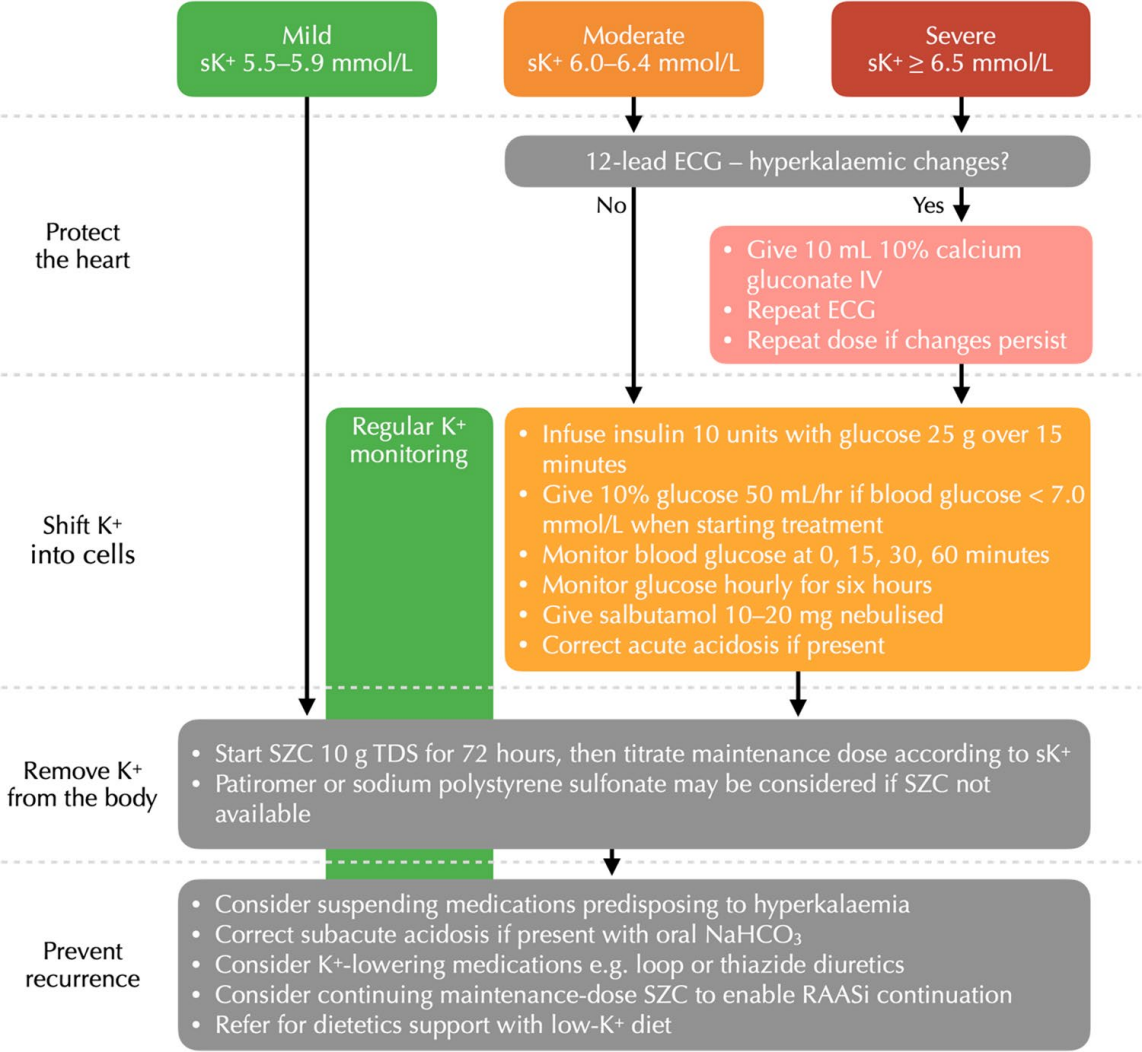
Suggested management algorithm for patients presenting with sK^+^  ≥ 5.5 mmol/L. ECG = electrocardiogram. IV = intravenous. RAASi = renin–angiotensin–aldosterone system inhibitor. sK^+^  = serum K^+^. SZC = sodium zirconium cyclosilicate. TDS = three times a day

Hyperkalaemia ≥ 6.0 mmol/L is a medical emergency which requires treatment to prevent severe cardiac arrhythmia. The hyperkalaemic patient must receive a 12-lead ECG to assess for hyperkalaemic changes and must undergo urgent sK^+^ measurement via both point-of-care and formal laboratory methods. The UK Renal Association recommends a five-step approach to managing acute hyperkalaemia: (i) protect the heart; (ii) shift K^+^ into cells; (iii) remove K^+^ from the body; (iv) monitor K^+^ and glucose; and (v) prevent recurrence (see Fig. 1) [63]. In the presence of hyperkalaemia-associated ECG changes, cardiac protection should be achieved by administration of intravenous calcium gluconate or calcium chloride. A repeat ECG should be performed 3–5 min after administration to assess for response, and a further dose of IV calcium considered if hyperkalaemic changes persist.

Infusion of 10 units insulin in 25 g glucose solution is recommended for patients presenting with sK^+^  ≥ 6.0 mmol/L to shift K^+^ to the intracellular compartment. Patients must be monitored for hypoglycaemia. The most common risk factors for treatment-induced hypoglycaemia include low pre-treatment blood glucose (i.e. < 7 mmol/L), in which situation a 50 mL/h 10% glucose infusion over five hours is recommended [63]. Insulin–glucose may be combined with either nebulised or IV salbutamol to increase intracellular shift of K^+^. The use of sodium bicarbonate in the management of hyperkalaemia in the acute setting is controversial. Some evidence suggests it is ineffective, while some studies demonstrate a potassium-lowering effect in patients presenting with combined hyperkalaemia and acidosis [64].

Removal of K^+^ from the body is recommended via the use of SZC in the first instance, which has a faster onset of action than patiromer and lower risk of binding to co-administered medications [50, 59, 65, 66]. Cation exchange resins such as calcium polystyrene sulfonate (Calcium Resonium®) are no longer recommended in the acute setting due to their slower onset of action.

## Management of Hyperkalaemia on RAASi Therapy

A patient who is receiving RAASi therapy may become hyperkalaemic but not meet criteria for acute treatment or admission. In this situation, clinicians will have to decide on a management strategy to manage the patient’s hyperkalaemia while maintaining as much medical benefit from RAASi therapy as possible. Medications with a potassium-lowering effect such as loop diuretics may be used if the patient’s fluid and electrolyte status are amenable. A recent study demonstrated that the thiazide diuretic chlorthalidone was effective at reducing blood pressure in CKD patients and also acted to reduce sK^+^ [67]. While the use of sodium bicarbonate is controversial in the acute setting, oral sodium bicarbonate may be used to aid correction of subacute hyperkalaemia in the context of subacute metabolic acidosis. Further to these medical therapies, patients should be encouraged to follow a low-K^+^ diet and should receive multidisciplinary support from dietetics services, where available.

## Conclusion

We are in an exciting chapter for the treatment of hyperkalaemia in HF. New therapies such as SZC and patiromer are not only contributing to the management of acute hyperkalaemia, but offer the potential to greatly improve access to life-saving RAASi therapies by tackling and preventing hyperkalaemia in the community. Data from HF trials now routinely report rates of hyperkalaemia (and often severe hyperkalaemia), but exact rates of hyperkalaemia-related adverse events such as arrhythmia, hospitalisation, or death are less well-reported. From the studies that have documented these details, however, we can see that in a clinical trial context, the risk of harm from RAASi-related hyperkalaemia is low, and the benefits of medical therapy outweigh the risks. A caveat is that participants in these trials have their sK^+^ closely monitored, and ensuring safety in a clinical context will likely entail close monitoring also. In addition, further research must be done into patients with HF in addition to other comorbidities. The ongoing LIFT study [68], which will examine the use of SZC to enable RAASi use in comorbid CKD-HF patients, is one such trial that will aim to address these patients’ needs.
